# Feline Testicular Biometry and Gonadosomatic Index: Associations Among Conventional Measurements, Mathematical Estimates, and Seminal Parameters

**DOI:** 10.3390/ani15152191

**Published:** 2025-07-25

**Authors:** Mónica Madrigal-Valverde, Rodrigo F. Bittencourt, Antonio Lisboa Ribeiro Filho, Thereza Cristina Calmon de Bittencourt, Isabella de Matos Brandão Carneiro, Luiz Di Paolo Maggitti, Gabriel Felipe Oliveira de Menezes, Carmo Emanuel de Almeida Biscarde, Gleice Mendes Xavier, Paola Pereira das Neves Snoeck, Larissa Pires Barbosa

**Affiliations:** 1 San Carlos Local Technological Campus—CTLSC, Costa Rica Institute of Technology, San Carlos 223-21001, Costa Rica; mmadrigal@itcr.ac.cr; 2 Área Académica Doctorado en Ciencias Naturales para el Desarrollo, San Carlos Local Technological Campus—CTLSC, San Carlos 223-21001, Costa Rica; 3 School of Veterinary and Animal Science Medicine, Federal University of Bahia, Av. Adhemar de Barros, 500, Salvador 40170-110, Bahia, Brazil; alisboafilho@ufba.br (A.L.R.F.); calmon@ufba.br (T.C.C.d.B.); isabella.brandao.c@hotmail.com (I.d.M.B.C.); lpmaggitti@yahoo.com.br (L.D.P.M.J.); gmenezes.vet@gmail.com (G.F.O.d.M.); cbiscarde@ufba.br (C.E.d.A.B.); gleicemxavier@gmail.com (G.M.X.); 4 Department of Agricultural and Environmental Sciences, State University of Santa Cruz, Ilhéus 45662-000, Bahia, Brazil; paolasnoeck@uesc.br; 5 Center for Agricultural, Biological, and Environmental Sciences (CCAAB), Federal University of Recôncavo of Bahia (UFRB), Cruz das Almas 44380-000, Bahia, Brazil; larissa@ufrb.edu.br

**Keywords:** testicular volume, urethral catheterization, biometry

## Abstract

The development of biometric techniques in domestic animals has greatly advanced scientific practices in wildlife research. The association between seminal characteristics and body and testicular biometry enables the selection of suitable breeders, though appropriate measurement techniques are required. The present study assessed differences among conventional methods and formulas for estimating testicular parameters. Testicular length, width, and thickness were measured using three methods in 13 adult male domestic cats. Testicular area, volume, and weight were estimated, from which the gonadosomatic index (GSI) was calculated. Sperm were collected using an alpha-2 adrenergic agonist and urethral catheterization, and characterized in terms of volume, vigor, total motility, progressive motility, concentration, plasma membrane integrity, and morphology. The three methods were consistent in terms of testicular area, volume, weight, and GSI. Moderate positive correlations were observed for testicular weight (r = 0.61, *p* < 0.05) and GSI (r = 0.58, *p* < 0.05). Testicular parameters showed strong positive correlations among each other (r > 0.80, *p* < 0.05). We observed a moderate positive correlation between head length and progressive motility (r = 0.65, *p* < 0.05). In conclusion, all testicular measurement and estimation techniques showed comparable performance. Therefore, testicular biometry is useful for selecting breeding males in feline conservation programs, wherein larger body biometrics are related to improved seminal and reproductive parameters.

## Simple Summary

The refinement of reproductive science in domestic models is useful for developing breeding programs for threatened species. To apply such biotechnologies in wild felines, the physical characteristics of the animals and behavior of gametes must first be verified in domestic cats. However, several instruments have been developed for measuring testicular characteristics; thus, researchers must determine the most appropriate instrument and whether the measurements are representative of reproductive capacity. The present study identified three suitable methods for testicular measurement and various formulas for estimating the physical parameters of the body and testes. In addition, the association between the biometrics and semen characteristics was verified. We concluded that physical characteristics can be used as the primary selection criteria in wild animals.

## 1. Introduction

Body biometry is a key tool for estimating weight, age, and specific species characteristics, with parameters such as body length and thoracic diameter exhibiting a strong correlation with live weight [1]. The relationship is more significant when associated with testicular biometry, whose variables, such as testicular volume and weight, are linked directly to androgen production and spermatogenesis in domestic cats [2]. Such measurements are essential for reproductive management programs in wild felines as they facilitate prediction of individual reproductive efficiency [3].

However, the application of biometric techniques in the evaluation of wild felines depends on standardized and reproducible measurement methods and formulas capable of guaranteeing the accuracy and effectiveness of the analyses [3,4]. Despite their importance, studies that explore body biometry, testicular biometry, and seminal parameters in felines in an integrated manner are still scarce, especially considering different testicular measurement methodologies [2,4].

Biometric techniques require methodological rigor and an understanding of the impact of the measurements and their applicability in different management contexts [4,5]. Free-living populations represent unique opportunities for collecting biometric data during capture for ecological studies and telemetric marking. In such a context, using body and testicular measurements as predictors of seminal quality can optimize the selection of individuals as genetic material donors [5,6].

Understanding the correlations between biometric measurements and seminal parameters would advance our basic reproductive biology knowledge, facilitating efficient application of assisted reproduction techniques [5]. In such a context, the domestic cat is a valuable experimental model because of its anatomical and physiological similarities with wild felines, in addition to the relative ease of handling and availability for studies [4,7].

Understanding of testicular biometry and its correlation with seminal parameters could provide insights that could facilitate the selection of appropriate males for assisted reproduction programs. In the present study, body and testicular biometry, as well as the differences among the methods and five formulas used for estimating testicular parameters were studied. This represents the first comparative record of the factors in the estimation of testicular biometry parameters in domestic cats.

## 2. Materials and Methods

### 2.1. Ethical Approval

The study was conducted in accordance with international ethical principles for animal research and was approved by the Institutional Animal Use Ethics Committee (protocol: 05–2022).

### 2.2. Location and Animal Assessment

All experiments were performed in 2018 at the Andrology Laboratory of the Veterinary Hospital at the Renato Rodenburg de Medeiros Neto da Federal University of Bahia (Androlab-Hospmev/UFBA), northeast Brazil (Salvador municipality, Bahia state, 12°58′16″ S, 38°30′39″ W). The study included 13 healthy mature male crossbred cats (referred to hereafter as tomcats) between 1 and 4 years of age with an average weight of 4.5 ± 0.4 kg. All animals were deemed healthy based on hematological and biochemical evaluations, followed by clinical and andrological examinations. All tomcats were privately owned, lived in apartments, fed cat food, and had no contact with other cats prior to the experimental period.

Serum testosterone secretion was confirmed by the presence of penile spicules. Testicular mobility and consistency within the scrotum were considered normal. Weight and age were homogeneous (*p* > 0.05).

### 2.3. Anesthetic Procedures and Semen Collection

Tomcats were administered an α-2 agonist in combination with ketamine for semen collection [7]. After 20 min, when muscle relaxation and no response to stimulation was observed, the penis of the tomcat was exposed and cleaned, and a sterile catheter without a side window (diameter: 1 mm, length: 13 cm; Provar^®^ kit, Provar, São Paulo, Brazil) was inserted carefully into the penile urethra up to 7 cm and left for ~40 s [7]. Semen entered the catheter via capillary action, after which the catheter was removed and semen was transferred to preheated 1.5 mL polypropylene tubes containing an isotonic 50 µL Tris–egg yolk diluent solution [7]. After semen collection, all animals underwent orchiectomy.

### 2.4. Body Biometry

Animals were placed in lateral recumbency, and a plastic measuring tape was used to measure head length (from snout to C1 vertebra), head circumference, head plus body length (from snout tip to S3 vertebra), tail length (cd1–cd15 vertebrae), total length (from snout to cd15 vertebra), and thoracic diameter (measured around the heart).

### 2.5. Testicular Biometry and Testicular Parameters

**Figure 1:**
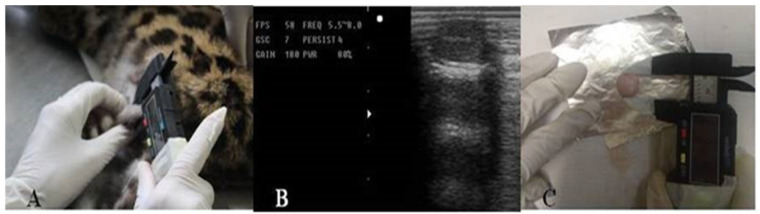
Methods for testicular measurements used to calculate testicular volume in adult felines. (**A**) Method 1: using a digital pachymeter before orchidectomy. (**B**) Method 2: ultrasound measurement. (**C**) Method 3: using a digital pachymeter after orchidectomy.

The testes, penis, and foreskin were inspected for ectoparasites and lesions. The testes were palpated for consistency and mobility within the scrotum and observed via ultrasonography (A5V; Sonoscape, Shenzhen, China) using a linear transducer at a frequency of 5.5–8 MHz. Testes height, breadth, and length were measured from the captured images (Method 2; Figure 1); the same measurements, along with the double skinfold of the scrotum [6], were performed using a digital caliper (LT-4237-000 Electronic Digital Caliper, Lufkin Industries, Missouri City, TX, USA) (Method 1; Figure 1). Bilateral orchiectomy was performed to remove the epididymis and testicular tunics, and measurements were conducted using a digital caliper (Method 3; Figure 1). Testes were weighed using an analytical balance according to Method 3 (Shimadzu Corporation, Kyoto, Japan).

The testicular area was estimated according to the area of an ellipse, that is

$$A=\pi r^{2}$$

Testicular volume was estimated using the following formulae:

Circle (used in [8]):

(1) $$VT=\frac{4}{3}\pi \;\frac{ABC}{2},$$

Howard et al. [9] and Villaverde et al. [2]:

(2) $$VT=\frac{4}{3}\pi \;\frac{\;A^{2}}{2}\times \frac{B}{2},$$

Lamberth’s formula [10]:

(3) VT=A×B×C×0.71,

Ellipsoid:

(4) VT=A×B×C×0.524,

Hansen [11]:

(5) $$VT=A\times B^{2}\times 0.52,$$

where *A* is the length, *B* is the height, and *C* is the width of the testes.

In all cases, testicular weight was estimated from the obtained volumes, with mammalian testicular density estimated at 1.046 g mL [12]. Testicular and total weights were used to estimate the GSI, as follows:

(6) $$\mathrm{GSI}=\frac{\mathrm{testicular}\;\mathrm{weight}\times 100}{body\;weight}.$$

#### Semen Analysis

Immediately after collection, all samples were sent to the laboratory and examined under a phase contrast microscope. Sperm vigor was estimated subjectively based on cell movement (scale: 0–5). Total motility (MT) and progressive motility (MP) were determined according to cell movement (0–100%) and also estimated subjectively (100× phase-contrast microscopy). Sperm concentration was determined using a Neubauer chamber, and the structural integrity of the sperm membrane was evaluated using the supravital test (EOS) [13]. The functional integrity of the plasma membrane was estimated using the hypo-osmotic test (HOST); full membrane functionality is observed in reactions with “folded tails.” Sperm morphology was analyzed in formol saline solution, and morphological alterations were observed using 100× phase-contrast microscopy and classified as either minor or major defects.

### 2.6. Statistical Analysis

Normality and homoscedasticity were verified using the Shapiro–Wilk and Levene tests, and measurements obtained by each method were used to estimate testicular area, volume, weight, and GSI.

To determine the agreement between the Method 1 measurements, testicular volume was estimated using Equations (1)–(5). The Bland–Altman method was used to estimate the means, limits of agreement, and confidence intervals: mean (vies); lower limit of agreement (LSC); upper limit of agreement (LIC). This is considered the standard statistical approach for assessing agreement between measurement methods and is commonly used in biomedical research [14]. The Bland-Altman method was also used for Methods 2 and 3 to assess agreement among clinical methods using Equations (1)–(5) for volumetric measurements. After that, the data were compared with each other using a *t*-test with a significance level of 95%.

When the equations were deemed adequate for each method, the agreement in the testicular volume results was determined using Equation (2) (Howard et al. [9] and Villaverde et al. [2]) and testicular weight and GSI were compared for all three methods. Associations between body biometry, testicular dimensions, and seminal variables were estimated using Pearson’s or Spearman’s linear correlations, based on the normality of the variables. All analyses were performed using SPSS version 13.0 (SPSS Inc., Chicago, IL, USA), with statistical significance set at *p* < 0.05. Data are presented as mean ± standard deviation (SD).

## 3. Results

Table 1 shows the mean ± standard deviation (SD) and median of testicular height, length, and width measurements in 13 tomcats. The dimensions were used to estimate testicular area and volume. The numerical differences obtained between the parameters studied, according to measurement methods (mm), did not differ significantly (*p* < 0.05). The measurements were used to estimate the area (one formula). For each method, the volume was estimated according to Equations (1)–(5) and testicular weight (Table 2).

Additionally, based on comparing the weight measurement obtained and that obtained on the scale. The comparison of Method 1 and the scale weight showed a standard deviation of 0.48. The standard deviation for Method 2 and the scale weight was 0.44, and the comparison of Method 3 and the scale weight was 0.68. These results were similar to or less than the standard deviation of the scale weight results. Based on this scientific criterion, agreement was established between the values.

It is noteworthy that in Method 3, compared to the scale weight, there is a difference between limits of 0.18.

Using the Bland–Altman analysis, the lower and upper limits of each pair of formulae were established (Table 3). Regarding our data set, those five formulae can be used interchangeably as the limits vary from in Method 1 between 0 and 0.31 cm^3^ (SD: 0–0.93), Method 2 between 0 and 0.60 cm^3^ (SD: 0–0.94), and Method 3 between 0 and 0.62 cm^3^ (0–1.09).

**Figure 2:**
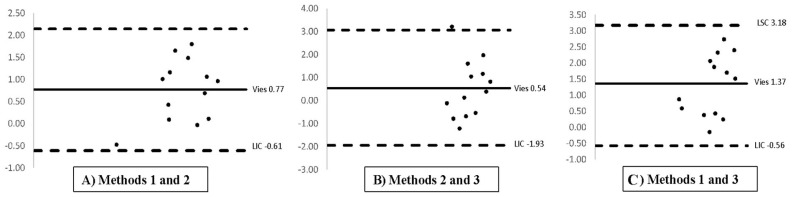
Bland-Altman analysis of agreement between methods for testicular area estimation. See Figure 1 for descriptions of the methods. Vies: mean; LSC: lower limit of agreement; LIC: upper limit of agreement.

The mean testicular areas ranged from 2.07 ± 0.46 to 3.37 ± 0.75 cm^2^. The formula used to calculate the elliptical area demonstrated strong agreement among the different measurement methods. Pairwise comparisons showed mean differences of 0.77 cm^2^ between Method 1 and Method 2, 0.54 cm^2^ between Method 2 and Method 3, and 1.37 cm^2^ between Method 1 and Method 3. All values fell within the limits of agreement (defined by −1.96 SD and +1.96 SD), as illustrated in Figure 2. The findings indicate good concordance among the three methods for estimating testicular area.

**Figure 3:**
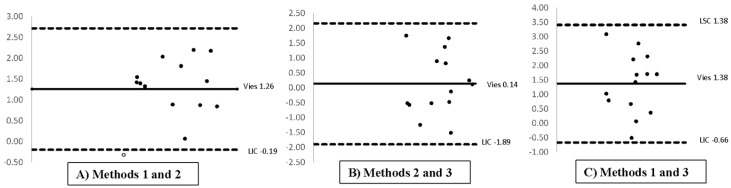
Bland-Altman analysis of agreement between equations for estimating testicular volume. See Figure 1 for the descriptions of the methods.

Regarding testicular volumes, the Bland–Altman analysis indicated that the distribution and confidence limits were similar across the five equations (*p* > 0.05, Table 3). The equations produced consistent results within and across methods, as illustrated in Figure 3. The average volumes were similar within each method, with mean differences of 1.26 cm^3^ between Methods 1 and 2, 0.14 cm^3^ between Methods 2 and 3, and 1.38 cm^3^ between Methods 1 and 3, according to Bland–Altman analysis.

In addition, the limits established by the Bland-Altman method were close, with this closeness between data being similar to the standard deviation of the mean volume values. This reduced differences in values and standard deviations for the five formulas in the three months and was concorded between formulas and measurement methods, Ireaffirming the indifferent use of these. We found no differences in the means when comparing the data with the *t*-test.

The equation selected for estimating testicular weight that was used to calculate the final mean and median values presented in Table 4 was Formula (2), which has been used to determine testicular volume in domestic cats (Howard et al. [9] and Villaverde et al. [2]), due to the indiscriminate use of the formulas. Its documented use for estimating testicular volume in felines and a detailed examination of the formula comparison data revealed that the difference between limits for Formula (2) and other formulas in Method 1 was 0 to 0.31, for Method 2 was 0 to 0.25, and for method 3 was 0 to 0.56. Additionally, when observing the values between limits for methods comparing Equation (2) and the other equations, we observe that the difference between Formulae (1) and (2) was 0 to 0.16, between Equations (2) and (3) and (2) and (5) was zero, between Equations (2) and (3) was 0.06 to 0.31, and between Formulae (2) and (4) was 0 to 0.16.

**Figure 4:**
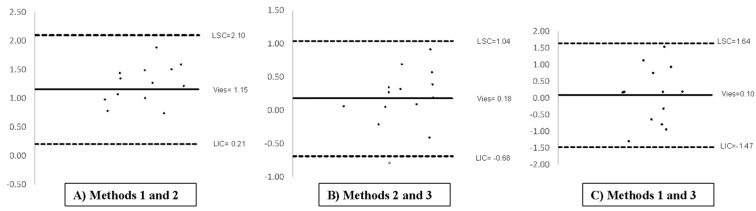
Bland-Altman analysis of agreement between testicular weight estimates and real weight scale. See Figure 1 for the descriptions of the methods.

To verify accuracy, the estimated volumes were compared to the actual testicular weights obtained using an analytical scale (Figure 4) according to the Bland–Altman analysis. This indicates good agreement between the estimated volumes and the actual weights, which has practical implications for future research and clinical applications.

We evaluated the reproductive potential of the tomcats through a semen analysis and investigated whether testicular or body biometrics correlate with seminal parameters. The descriptive statistics of the seminal parameters are shown in Table 5.

The descriptive statistics for body biometrics in adult domestic tomcats are presented in Table 6. These parameters were analyzed to investigate potential associations with testicular dimensions and seminal characteristics.

Strong positive linear correlation (Spearman’s r = 0.90, *p* < 0.05) between tail and total lengths, moderate positive correlation between head circumference and total length (r = 0.70, *p* < 0.05), and moderate positive correlation between head length and circumference (r = 0.72, *p* < 0.05) were verified. Testicular volume, weight, and GSI exhibited strong positive correlations between these parameters (r ≥ 0.90, *p* < 0.05). For Methods 1 and 2, r = 0.56 and *p* < 0.05 for GI for the fifth equation.

For body biometry, strong positive correlations were observed between tail length with head circumference (r = 0.72, *p* < 0.05) and total length (r = 0.90, *p* < 0.05). Moreover, body length was significantly correlated (*p* < 0.05) with testicular parameters.

In addition, strong positive correlations were observed between sperm vigor and total motility (r = 0.86, *p* < 0.05), sperm vigor and progressive motility (r = 0.75, *p* < 0.05), total motility and progressive motility (r = 0.98, *p* < 0.05), sperm vigor and EOS (r = 0.84, *p* < 0.05), total motility and EOS (r = 0.75, *p* < 0.05), and progressive motility and EOS (r = 0.72, *p* < 0.05) (Table 7).

In Methods 2 and 3, no significant correlations (*p* > 0.05) were observed between body and testicular biometry. In Method 1, moderate positive correlations were observed between volume and testicular weight and tail length (r = 0.64, *p* < 0.05), as well as between the same testicular parameters and total length (r = 0.76, *p* < 0.05).

Sperm concentration had a moderate positive correlation (r = 0.71, *p* < 0.05) with testicular parameters (volume and weight) for Method 1. Furthermore, a moderate positive correlation was observed between head length and progressive motility (r = 0.65, *p* < 0.05).

## 4. Discussion

According to the results of the present study, no differences were observed among the five equations used. In literature, no consensus is available on the type of equation used for testicular measurements in all animal species or specifically for felines. Therefore, the information generated in the present investigation allows researchers to confidently process testicular data using one equation or another.

The mean testicular volume and weight in the present study were similar to those reported by Villaverde et al. [2], who used Formula (2) and found averages of 2.93 ± 0.6 cm^3^ and 2.71 ± 0.7 g. Similarly, using B-mode ultrasonography, Stremnitzer [15] reported a mean volume of 1.8 cm^3^ and a testicular length of 1.9 cm—values comparable to those obtained in the present study using the same technique.

Several studies have highlighted variations in testicular volume depending on the methodology employed. Applying the water displacement method, Kumar et al. [16] reported volumes ranging from 6.33 ± 0.25 cm^3^ to 6.85 ± 0.47 cm^3^ across six samples. Homola et al. [17], evaluating 33 cats, found smaller volumes (0.77–0.97 cm^3^), depending on the measurement tool (Podany ruler or ultrasonography with Formula (1)). The authors also identified significant differences between methods, likely because of inclusion of epididymal structures in the measurements. Such discrepancies may be explained based on methodological differences, age, body weight, seasonality, and animal management conditions.

Despite methodological variability, the present study demonstrated that manual techniques (in vivo and in vitro) and ultrasonography yielded estimates consistent with actual testicular weight. This is supported by the Bland–Altman analysis results, which indicated no significant bias (*p* > 0.05). Thus, the tested methods are reliable for estimating testicular volume and may be suitable for selecting breeding animals.

Considering that testicular volume estimates obtained via caliper and ultrasound are equally valid [18], the volumes estimated through ultrasonography showed strong correlation with both actual volumes and those estimated using the other techniques applied in the present study (r = 0.853–0.871; *p* = 0.0001).

Non-invasive methods for testicular measurement are beneficial in clinical and reproductive settings, especially in wildlife species. Ultrasonography offers the additional advantage of enabling assessment of parenchymal integrity. Nevertheless, using calipers remains a practical and accessible alternative in resource-limited environments. In general, the use of Methods 1 and 2 eliminates the effects of removal of the scrotum and its folds on testicular measurements, as verified for the width, which is numerically greater in all animals with Method 3, although the difference is not significant (*p* > 0.05). The five formulae used to estimate testicular volume and weight had similar results (*p* > 0.05). Considering the lack of significant differences, Formula (2), which has been studied widely and used to determine testicular volume in domestic cats (Howard et al. [9] and Villaverde et al. [2]), is recommended for use.

The volume of semen collected via urethral catheterization was consistent with previously reported values: 20.9 ± 15.1 μL [19] and 21.1 ± 31.9 μL [20]. Jelinkova et al. [20] described a wide range (2.0–185.0 μL), which is consistent with the findings of this study.

Total and progressive sperm motility observed in the present study were higher than those reported by Filliers et al. [21] and within the range described by Prochowska et al. [22] (25–90%). The mean progressive motility also exceeded the maximum values reported by Jelinkova et al. [20] for urethrally collected samples (maximum of 75%).

The high motility and sperm integrity likely explain the greater proportion of morphologically normal sperm observed in the present study. The values surpassed those reported in previous studies (57.5 ± 9.6% [22], 58.27 ± 17.8% [21]), although wide variability is reported in the literature, from 1.0% to 82.0% [20], a range also observed in the present data (see Table 6).

In wild felids, positive correlations have been observed between body biometrics and testicular weight [8]. A similar pattern was observed in the present study with domestic cats, where total body length was significantly correlated (*p* < 0.05) with testicular parameters. Therefore, body biometric measurements may be criteria applicable for breeding males. Animals with greater total body length had larger testes, translating into greater testicular area, volume, weight, and GSI.

The GSI, which represents the proportion of body mass allocated to the gonads, is a key indicator of spermatogenic capacity and male reproductive status. It also serves as a metric for assessing environmental and nutritional impacts on gonadal function. GSI values vary according to species body size and reproductive behavior, with smaller polyandrous species generally exhibiting higher GSI values [23,24]. For example, while the jaguar (Panthera onca) exhibits a GSI of 0.034 [18], smaller felids such as the jaguarundi (*Herpailurus yagouaroundi*) and the margay (*Leopardus wiedii*) show higher values of 0.10 and 0.09, respectively [7]. The domestic cat, in turn, allocates approximately twice as much gonadal mass as observed in jaguars or pumas [25].

The mean GSI observed in the present study (0.05–0.07) is consistent with previously reported values for the species [25] and comparable to those observed in large felids, such as the jaguar [18]. Moreover, this index was correlated with body measurements and sperm concentration, reinforcing its potential as a reproductive assessment tool [2]. Further research exploring the use of GSI could provide valuable insights into reproductive health and the ecological factors affecting fertility, contributing significantly to conservation efforts.

Testicular volume is considered one of the leading reproductive potential indicators because of its direct correlation with testicular weight and spermatogenic capacity. It reflects the number of seminiferous tubules and the number of Sertoli and germ cells, as confirmed by histological analyses in association with testicular measurements [8,25]. Although the relationship is well established in humans and various domestic species, data on domestic cats remain scarce, highlighting the relevance of the findings presented here.

In the present study, testicular biometric parameters were positively correlated with sperm concentration and motility. Animals with heavier and larger testes exhibited higher sperm concentrations and better progressive motility. Similarly, Villaverde et al. [2] reported a positive association between testicular volume and semen quality, supporting the use of the measurements as indicators of spermatogenic efficiency and reproductive fitness. Also, a moderate positive correlation was observed between head length and progressive motility (r = 0.65, *p* < 0.05), so that head length is a physical parameter that could be used for the selection of felines for semen collection.

## 5. Conclusions

In summary, in the present study, manual and ultrasonographic methods proved effective for estimating testicular dimensions and weight in domestic cats. Caliper-based measurements offer a practical and accessible tool for field applications, whereas ultrasonography provides added diagnostic value by enabling the assessment of testicular parenchymal integrity and overall reproductive health.

The correlations observed between body and testicular biometrics and key sperm parameters underscore the usefulness of these measurements for estimating reproductive potential. These associations can guide the selection of high-quality breeding males and support the development of specific reproductive strategies.

Therefore, this study should be replicated in domestic cats by selecting breeding males classified according to their physical characteristics (head length or testicular parameters) and their association with sperm parameters. This line of research ultimately seeks to extrapolate this to wild cats.

## Figures and Tables

**Table 1:** Central tendency measures of testicular height, width, and length in tomcats assessed using different measurement methods (mm).

	Method 1			Method 2			Method 3		
	Length	Height	Width	Length	Height	Width	Length	Height	Width
Mean ± SD (mm)	14.42 ± 3.89	7 ± 3.89	9.31 ± 3.89	17.14 ± 2.54	12.28 ± 1	10.2 ± 0.86	15.15 ± 1.49	12.42 ± 1.26	22.56 ± 2.78
Median (mm)	14.7	9.3	9.3	18	18	10	15.5	12.6	21.5

Method 1: digital pachymeter before orchidectomy. Method 2: ultrasound measurement. Method 3: digital pachymeter after orchidectomy. *p* > 0.05.

**Table 2:** Mean ± standard deviation for the three methods used for testicular measurements, as well as for each formula used to calculate area (cm^2^), volume (cm^3^), weight (g), and real weight (affected by the scale in grams).

			Method 1						Method 2						Method 3				Real	
Equation	Testicular Area		Testicular Volume		Testicular Weight		Testicular Area		Testicular Volume		Testicular Weight		Testicular Area		Testicular Volume		Testicular Weight		Scale Testicular Weight	
(1)	2.10	±0.47	4.45	±0.72	4.65	±0.75	3.77	±0.49	2.60	±0.84	2.72	±0.88	2.24	±1.03	9.00	±2.62	9.42	±2.74	2.16	±0.67
(2)	2.10	±0.47	1.30	±0.43	1.36	±0.45	3.77	±0.49	2.46	±0.61	2.57	±0.64	2.24	±1.03	2.87	±0.83	3.00	±0.87	2.16	±0.67
(3)	2.10	±0.47	1.51	±0.24	1.58	±0.25	3.77	±0.49	0.88	±0.29	0.88	±0.29	2.24	±1.03	1.57	±0.37	1.64	±0.39	2.16	±0.67
(4)	2.10	±0.47	1.12	±0.18	1.11	±0.30	3.77	±0.49	0.65	±0.21	1.11	±0.30	2.24	±1.03	1.16	±0.27	1.11	±0.30	2.16	±0.67
(5)	2.10	±0.47	1.34	±0.22	1.40	±0.23	3.77	±0.49	0.65	±0.21	0.68	±0.22	2.24	±1.03	1.27	±0.28	1.33	±0.29	2.16	±0.67

Method 1: digital pachymeter before orchidectomy. Method 2: ultrasound measurement. Method 3: digital pachymeter after orchidectomy. Testicular volume was estimated using the following formulae: Circle: VT = 4/3 π ABC/2, (1); Howard et al. [9] and Villaverde et al. [2]: VT = 4/3 π (A^2^)/2 × B/2, (2); Lamberth’s formula [10]: VT = A × B × C × 0.71, (3); Ellipsoid: VT = A × B × C × 0.524, and (4); Hansen [11]: VT = A × B^2^ × 0.52, (5), where A is the length, B is the height, and C is the width of the testes. *p* > 0.05.

**Table 3:** Mean differences (± standard deviation) with their respective upper and lower confidence limits (LSC to LIC), for each formula comparison within each method of testicular volume estimation in tomcats.

	Method 1			Method 2			Method 3		
Formulae	Mean (cm^3^) ± (SD)		Absolute Differences Between Limits (cm^3^)	Mean (cm^3^) ± (SD)		Absolute Differences Between Limits (cm^3^)	Mean (cm^3^) ± (SD)		Absolute Differences Between Limits (cm^3^)
1 and 2	−0.01	±−0.01	0.00	0.23	±−0.27	0.04	0.55	±−0.39	0.16
(LSC to LIC)	(−0.76	to −0.17)	0.59	(−0.31	to 0.76)	0.32	(−0.21	to 1.30)	1.09
1 and 3	0.46	±−0.15	0.31	0.79	±−0.19	0.60	−0.82	±−0.20	0.60
(LSC to LIC)	(0.17	to 0.76)	0.59	(0.42	to 1.16)	0.74	(−0.95	to 0.36)	0.59
1 and 4	0.00	±−0.00	0.00	0.00	±−0.00	0	0.00	±−0.00	0
(LSC to LIC)	(−0.76	to −0.17)	0.59	(0.00	to 0.00)	0	(0.00	to 0.00)	0
1 and 5	−0.01	±−0.01	0.00	0.21	±−0.27	0.05	0.53	±−0.39	0.16
(LSC to LIC)	(−0.76	to −0.17)	0.59	(−0.32	to 0.74)	0.32	(−0.22	to 1.28)	1.06
2 and 3	0.46	±−0.15	0.31	0.57	±−0.32	0.25	0.28	±−0.34	0.06
(LSC to LIC)	(0.17	to 0.76)	0.59	(−0.06	to 1.20)	0.06	(−0.38	to 0.94)	0.56
2 and 4	0.00	±−0.01	0.00	−0.22	±−0.27	0.05	−0.55	±−0.39	0.16
(LSC to LIC)	(−0.76	to −0.17)	0.59	(−0.76	to 0.31)	0.31	(−1.30	to 0.21)	1.09
2 and 5	−0.01	±−0.00	0.00	−0.02	±−0.00	0	0.00	±−0.01	0
(LSC to LIC)	(−0.01	to 0.00)	0.01	(−0.03	to −0.01)	0.03	(0.00	to 0.00)	0
3 and 4	−0.46	±−0.15	0.31	−0.79	±−0.19	0.60	(−0.82	±−0.20	0.62
(LSC to LIC)	(−0.78	to −0.17)	0.59	(−1.16	to −0.42)	0.41	−0.95	to 0.36)	−0.59
3 and 5	−0.47	±−0.15	0.31	−0.59	±−0.32	0.27	(−0.30	±−0.33	0.03
(LSC to LIC)	(−0.78	to −0.17)	0.59	(−1.21	to 0.04)	1.16	−0.95	to 0.36)	0.59
4 and 5	−0.01	±−0.00	0.00	0.21	±−0.27	0.06	(0.53	±−0.39	0.14
(LSC to LIC)	(−0.02	to 0.00)	0.02	(−0.32	to 0.74)	0.42	−0.22	to 1.27)	1.05

Formulae: 1 = circle volume; 2 = Howard et al. [9] and Villaverde et al. [2]; 3 = Lamberth’s formula [10]; 4 = ellipse volume; 5 = Hansen [11]. LSC: lower limit of agreement; LIC: upper limit of agreement.

**Table 4:** Measures of central tendency for testicular measurements in domestic tomcats.

	Area (cm^2^)		Volume (cm^3^)		Weight (g)		GSI	
	Mean ± SD	Median	Mean ± SD	Median	Mean ± SD	Median	Mean ± SD	Median
Method 1	2.10 ± 0.47	2.00	1.30 ± 0.43	1.22	1.36 ± 0.45	1.28	0.03 ± 0.01	0.03
Method 2	3.77 ± 0.49	3.68	2.46 ± 0.61	2.28	2.57 ± 0.64	2.38	0.06 ± 0.01	0.06
Method 3	2.24 ± 1.03	2.93	2.87 ± 0.83	2.94	3.00 ± 0.87	3.08	0.07 ± 0.02	0.07

GSI: gonadosomatic index. Method 1: digital pachymeter before orchidectomy. (B) Method 2: ultrasound measurement. (C) Method 3: digital pachymeter after orchidectomy. Testicular volume used Equation (2) (Howard et al. [9] and Villaverde et al. [2]). *p* > 0.05.

**Table 5:** Descriptive statistics of seminal parameters.

Variable	Volume (μL) *	Vigor *	MT (%)	MP (%)	CN/mL *	EOS (%)	CH (%)	EMN (%)
Mean ± DP	37.50 ± 28.41	3.85 ± 0.41	81.50 ± 8.51	75.00 ± 10.27	473.31 ± 491.61	78.60 ± 11.16	80.70 ± 9.01	67.00 ± 20.74
Minimum	20.00	3.00	70.00	60.00	10.00	64.00	66.00	31.00
Q25	20.00	3.50	73.75	67.50	41.52	68.75	70.00	50.75
Median	30.00	4.00	80.00	75.00	323.90	77.50	84.50	68.50
Q75	37.50	4.00	90.00	85.00	881.25	88.50	88.25	83.50
Maximum	115.00	4.50	95.00	90.00	1333.33	95.00	90.00	96.00

* Non-normal variables. MT (%): total motility; MP (%): progressive motility; CN: sperm concentration; EOS (%): membrane structural integrity; CH (%): membrane functional integrity; EMN (%): normal sperm morphology.

**Table 6:** Descriptive statistics for body biometrics in adult domestic tomcats.

Measurement (cm)	Mean ± SD (cm)	Minimum (cm)	Q25	Median (cm)	Q75	Maximum (cm)
Head length	12.58 ± 1.17	11.00	11.75	12.00	13.50	15.00
Head circumference	23.04 ± 1.75	20.00	21.75	23.00	24.00	27.00
Thoracic diameter	32.54 ± 1.51	30.00	32.00	32.50	33.00	35.50
Tail + body lengths	55.77 ± 1.52	53.00	54.75	56.00	57.00	58.00
Tail length	27.38 ± 4.58	14.50	25.75	28.00	30.25	33.00
Total length	83.15 ± 4.72	70.50	81.50	83.00	86.75	89.50

**Table 7:** Spearman’s correlations for body biometry and sperm parameters.

	Tail + Body Lengths	Tail Length	T. Diameter	Head Circ	Total Length	Head Length	Sp.Vol	Vigor	TM	PM	Cn	EOS	CH	MA
Tail + body lengths	1.00	−0.11	0.00	−0.06	0.32	0.28	0.13	0.55	0.50	−0.03	−0.26	0.08	−0.57	−0.25
Tail length	−0.11	1.00	0.28	0.72 *	0.90 **	0.04	−0.03	−0.36	−0.26	0.22	0.51	0.21	−0.15	0.55
T. diameter	0.00	0.28	1.00	0.51	0.26	−0.12	−0.23	−0.12	−0.15	0.05	0.17	0.21	−0.29	−0.03
Head circ	−0.06	0.72 *	0.51	1.00	0.70 *	−0.14	−0.31	−0.54	−0.33	0.07	0.70 *	0.29	−0.18	0.43
Total length	0.32	0.90 **	0.26	0.69 *	1.00	0.12	−0.08	−0.18	−0.13	0.17	0.44	0.24	−0.45	0.47
Head length	0.28	0.04	−0.12	−0.14	0.12	1.00	0.46	0.66 *	0.57	0.65 *	−0.02	0.30	0.41	0.39
Vol	0.13	−0.03	−0.23	−0.31	−0.08	0.46	1.00	0.74*	0.72 *	−0.13	-0.677 *	−0.56	0.22	0.18
Vigor	0.55	−0.36	−0.12	−0.54	−0.18	0.658 *	0.74 *	1.00	0.86 **	0.75 *	-0.698 *	0.75*	0.02	−0.13
TM	0.50	−0.26	−0.15	−0.33	−0.13	0.57	0.72 *	0.86 **	1.00	0.95 **	−0.49	−0.28	0.18	−0.24
PM	−0.03	0.22	0.05	0.07	0.17	0.65 *	−0.13	0.75 *	0.95 **	1.00	0.49	0.72 *	0.56	0.22
Cn	−0.26	0.51	0.17	0.695 *	0.44	−0.02	−0.68	−0.70	−0.49	0.49	1.00	0.60	0.17	0.36
EOS	0.08	0.21	0.21	0.29	0.24	0.30	−0.56	0.75 *	−0.28	0.72 *	0.60	1.00	0.19	0.02
CH	−0.57	−0.15	−0.29	−0.18	−0.45	0.41	0.22	0.02	0.18	0.56	0.17	0.19	1.00	0.16
Sp.Vol	−0.25	0.55	−0.03	0.43	0.47	0.39	0.18	−0.13	−0.24	0.22	0.36	0.02	0.16	1.00

* *p* < 0.01 ** *p* < 0.05. T. diameter: thoracic diameter; Head circ: head circumference; Sp.Vol: sperm volume (ml); MT (%): total motility; MP (%): progressive motility; CN: sperm concentration; EOS (%): membrane structural integrity; CH (%): membrane functional integrity; M (%): sperm morpho-anomalies.

## Data Availability

The original contributions presented in the study are included in the article. Further inquiries can be directed to the corresponding author.
